# SMAD4 is a predictive marker for 5-fluorouracil-based chemotherapy in patients with colorectal cancer

**DOI:** 10.1038/sj.bjc.6600511

**Published:** 2002-09-09

**Authors:** J-L Boulay, G Mild, A Lowy, J Reuter, M Lagrange, L Terracciano, U Laffer, R Herrmann, C Rochlitz

**Affiliations:** Department of Research, University Hospital, CH-4031 Basel, Switzerland; The Swiss Group for Clinical Cancer Research (SAKK), CH-3008 Bern, Switzerland; Department of Pathology, University Hospital, CH-4031 Basel, Switzerland; Department of Oncology, University Hospital, CH-4031 Basel, Switzerland

**Keywords:** colorectal cancer, TGFβ, signalling, SMAD, predictive marker

## Abstract

The gene for the transducer of transforming growth factor-beta/bone morphogenetic protein signalling SMAD4, a potential suppressor of colorectal carcinogenesis, is located at the chromosomal region 18q21. In order to evaluate the clinical relevance of *SMAD4* deletion, gene copy alterations were determined by copy dosage using real-time quantitative PCR in 202 colorectal tumour biopsies from a previous randomised study of adjuvant chemotherapy. Patients with normal *SMAD4* diploidy turned out to have a three-fold higher benefit of 5-fluorouracil-based adjuvant chemotherapy with a border line significance (overall survival: 3.23, *P*=0.056; disease-free survival: 2.89, *P*=0.045). These data are consistent with the previous observation that patients whose cancer had retention of the 18q21 region had a significantly higher benefit from 5-fluorouracil-based therapy. Moreover, these results may provide a refinement at the gene level of the clinical relevance of 18q21 deletion, thereby suggesting SMAD4 as a predictive marker in colorectal cancer. This data also indicate that integrity of this component of the transforming growth factor-beta/bone morphogenetic protein signalling pathway may be a critical factor for benefit of chemotherapy in patients with colorectal cancer.

*British Journal of Cancer* (2002) **21**, 630–634. doi:10.1038/sj.bjc.6600511 www.bjcancer.com

© 2002 Cancer Research UK

## 

Deletion of the chromosomal region 18q21 is the most frequent cytogenetic alteration observed in colorectal cancer (CRC), suggesting the location of a tumour suppressor locus in this region (Vogelstein *et al*, 1988; Mitelman *et al*, 1997). Searches for such candidate genes have led to the identification of a gene designated as *Deleted in Pancreatic Cancer locus 4* (*DPC4*) (Hahn *et al*, 1996; Thiagalingam *et al*, 1996). Through its homology with the *C. elegans* *Small* (*Sma*) proteins (Savage *et al*, 1996) and with the *Drosophila* protein *Mothers against dpp* (*Mad*), initially identified for its genetic interaction with the gene for the BMP-like peptide *Decapentaplegic* (*dpp*) (Raftery *et al*, 1995), *DPC4* has been renamed *SMAD4* as a merger of *Sma* and *Mad* (Derinck *et al*, 1996).

SMADs form a family of structurally related proteins initially identified for their role in embryonic development of *Drosophila* (Raftery *et al*, 1995) and of *C. elegans* (Savage *et al*, 1996). Proteins of the SMAD family can be divided into three distinct subtypes that correlate with their respective functions in transforming growth factor beta (TGFβ)/bone morphognetic protein (BMP) signalling (Kretschmar and Massagué, 1999; Newfeld *et al*, 1999), as depicted in Figure 1

**Figure 1:**
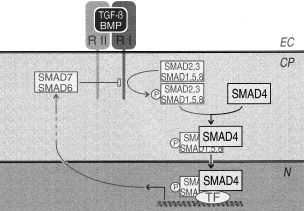
SMADS in the TGFβ/BMP signalling pathway: (i) receptor-activated (ra)-SMADs SMAD2 or SMAD3 are serine-phosporylated upon TGFβ-receptor interaction, whereas SMAD1, SMAD5 or SMAD8 phosphorylation is exclusively induced by BMPs; (ii) SMAD4, the common and unique co-SMAD signalling mediator to all TGFβ/BMP cytokines, heteropolymerises with activated ra-SMADs and migrates to the nucleus where it associates with tissue specific transcription factors (TF); (iii) anti-SMADs produced upon cytokine induction (SMAD7 for TGFβ and SMAD6 for BMPs) block ra-SMAD serine phosphorylation. This inducible negative feedback loop provides a transient response to cytokine activation (see Kretschmar and Massagué, 1999). Extracytoplasmic (EC), cytoplasmic (CP) and nuclear (N) compartments are indicated.

: (i) receptor-activated (ra)-SMADs are serine-phosporylated upon binding of the cytokine to its cognate receptor. SMAD2 and SMAD3 are specifically activated by TGFβ-like cytokines, whereas SMAD1, SMAD5 and SMAD8 are exclusively phosphorylated by BMPs; (ii) a co-SMAD, SMAD4 heteropolymerises with activated ra-SMADs. This complex migrates to the nucleus where it associates with tissue specific transcription factors. SMAD4 is the only co-SMAD protein known in mammalians, and therefore is a common signalling mediator to all TGFβ/BMPs; and (iii) among the immediate target genes for SMAD transcription complexes are the genes for anti-SMADs. Thus, the anti-SMADs SMAD6 and SMAD7 prevent activation of ra-SMADs (SMAD1/5/8 and SMAD2/3, respectively), therefore providing a transient cytokine response through a negative feedback loop. Interestingly, SMAD2 (Eppert *et al*, 1996) and SMAD7 (Nakao *et al*, 1997) genes have also been assigned to the 18q21 region (Eppert *et al*, 1996; Röijer *et al*, 1998), where the SMAD7 gene maps between SMAD2 and SMAD4 genes (Boulay *et al*, 2001) within four megabases (Venter *et al*, 2001). Thus, this region encodes the three classes of TGFβ mediators specifically required for the signalling of TGFβ-like cytokines, and one, SMAD4, for both TGFβ and BMP families.

Genetic evidence for the involvement of TGFβ pathway in colon tumour suppression was given by Markowitz *et al* (1995), who observed frequent frameshift mutations within the TGFβ-receptor II coding sequence in CRC, as a result of microsatellite instability. This observation has been later confirmed in a larger population, where most tumors with microsatellite instability carry this gene mutation (Watanabe *et al*, 2001). On the other hand, TGFβ has been shown to be a potent cell growth inhibitor (Roberts and Sporn, 1993), and apoptosis inducer on prostatic epithelial cell lines (Hsing *et al*, 1996), whereas most squamous carcinoma lines are refractory to this function (Reiss *et al*, 1993; Blobe *et al*, 2000). Thus, the frequent deletion of the chromosomal region 18q21 in colorectal tumours together with the physiologic functions of TGFβ strongly suggested a role for SMAD4 in the suppression of colorectal carcinogenesis.

For these reasons, we wished to study the influence of the *SMAD4* gene on the clinical outcome of patients with CRC, including on benefit of 5FU-based chemotherapy. Indeed, an interaction between markers and treatment responsiveness or lack thereof has led to a separation of these factors into prognostic (independent of treatment) and predictive (interactive with treatment) categories. To do so, we took advantage of archived colorectal tumour biopsies collected in a previous Swiss Association for Clinical Cancer Research (SAKK) study of 5FU-based perioperative adjuvant therapy (SAKK, 1995). Through a strategy based on quantitative real time PCR (Boulay *et al*, 1999, 2001), we performed genetic analyses of corresponding DNAs by copy dosage of the *SMAD4* gene. In order to study on one hand the prognostic value of genotype, and on the other hand its predictive effect on the efficacy of 5FU-based therapy among patients with CRC, we undertook multivariate statistical analysis of *SMAD4* gene copy status on survival.

## MATERIALS AND METHODS

### Patients

Patients from whom biopsies were isolated, were part of a previous randomised study of the Swiss Association for Clinical Cancer Research (SAKK) on benefit of adjuvant chemotherapy (SAKK study 40/81) (SAKK, 1995). In that study, 533 patients with colorectal cancer about to undergo curative resection were randomly assigned no adjuvant treatment (control group) or an immediate postoperative infusion with 5FU (500 mg m^−2^) for 7 days, with one single dose of mitomycin (10 mg m^−2^) on day 1. As a result, patients appeared to significantly benefit from this therapy such that overall survival increased from 55 to 66 months (hazard ratio: 0.74; 95% confidence interval: 0.57–0.97; *P*=0.026), and disease-free survival, from 48 to 57 months (hazard ratio: 0.79; 95% confidence interval: 0.62–1.00; *P*=0.051). The relationship between genotypes and clinical outcome was assessed in a subset of 202 patients with genetic data for which we also had clinical and survival data. As shown in Table 1

**Table 1:**
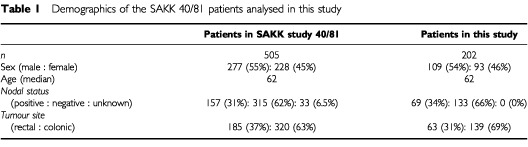
Demographics of the SAKK 40/81 patients analysed in this study

, the subgroup for which genetic and clinical data are available, is closely representative of the patients treated in the SAKK study 40/81 (SAKK, 1995). Our study comprises 164 out of the 233 individuals described in our previous report (Boulay *et al*, 2001).

### Gene copy status scoring

Genomic samples were tested for gene dosage using the TaqMan® system on an ABI Prism® 7700 sequence detector (PE Applied Biosystems, Foster, USA). All reactions were made in triplicate. For each individual, the Ct value (calculated by the built-in software) obtained for the gene *36B4* (Masiakowski *et al*, 1982) on normal tissue was subtracted from that of tumour tissue thus defining ΔCt36B4. A similar calculation was made for the *SMAD4* gene (ΔCtSMAD4). Gene copy status is indicated by the ΔCt value (ΔCt36B4–ΔCtSMAD4) as following. ΔCt>−0.45: no deletion; ΔCt<−0.55: hemizygous. Primers: *SMAD4*, gca gac aga aac tgg att aaa aca att and gaa tgt gtt tct cct aat ctt caa gct; *36B4*: agc aag tgg gaa ggt gta atc c and cca ttc tat cat caa cgg gta caa. Probes: *SMAD4*, tgt tgt ggt ccc tat ggc tgt tta cta tcc a; *36B4*: tct cca cag aca agg cca gga ctc g.

### Statistical analysis

Cox proportional hazard modelling was undertaken to assess the impact of genotype on overall survival and on disease-free survival after controlling for possible confounding. All analyses were performed using S Plus. The relationship between genotypes and survival/disease-free survival was assessed in a subset of 202 patients with genetic data for whom we also had clinical and survival data.

## RESULTS

We aimed to test whether the deletion of the *SMAD4* gene would have a significant influence on the outcome of patients with CRC. Within the individual tumours in this statistical analysis, the frequency of *SMAD4* gene deletion was 67% (135 out of 202). This deletion frequency was similar to that previously reported (Boulay *et al*, 2001). For each individual, the associations between gene copy dosage and clinical data was investigated in multivariate statistical analyses that included age, sex, stage, tumour location, grade, nodal status and chemotherapy as covariates. Hazard ratios (HR) for death and relapse associated with *SMAD4* gene deletion were close to one (1.22 and 1.16, respectively) with non-significant *P* values (0.43 and 0.56, respectively, Table 2

**Table 2:**
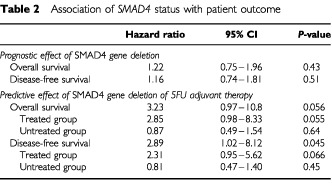
Association of *SMAD4* status with patient outcome

, top). Thus we concluded that deletion of *SMAD4* gene has no significant influence on the outcome of patients with CRC.

**Figure 2:**
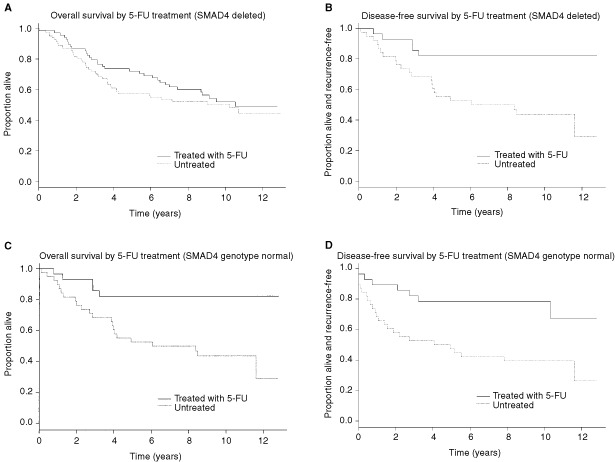
Kaplan–Meier plotting of survival in response to 5FU therapy in patients (*n*=202) with *SMAD4* deletion (top) and with no loss of *SMAD4* (bottom). Overall survival (left): HR=3.23, 95% CI=0.97–10.8, *P*=0.056. Disease-free survival (right): HR=2.89, 95% CI=1.02–8.12, *P*=0.045.

The original clinical study from which tumour samples were derived had shown the benefit of 5FU-based perioperative adjuvant chemotherapy in colorectal cancer (SAKK, 1995). Thus, a similar multivariate statistical analysis was performed to evaluate the deletion of the *SMAD4* gene as a potential marker for a predictive effect on 5FU treatment. Regarding disease-free survival, after controlling for confounding in the multivariate models, the HR associated with 5FU treatment among patients with normal diploidy for *SMAD4* was 0.32, whereas the HR associated with 5FU treatment in patients with *SMAD4* deletion was 2.89 times as large (Table 2, bottom). The difference between these two HRs (i.e. the statistical interaction between gene deletion and the effect of 5FU chemotherapy) was of borderline statistical significance (*P*=0.045). A similar result was found for overall survival, the HR associated with 5FU treatment being 0.25 among patients with normal *SMAD4* genotype and 3.23 times as great among patients with *SMAD4* deletion (Table 2, bottom; Figure 2

). As with disease-free survival, the difference was of borderline statistical significance (*P*=0.056). This suggests *SMAD4* as a predictive marker for 5FU/mitomycin adjuvant chemotherapy

## DISCUSSION

We established that among the patients with colorectal cancer involved in this study, *SMAD4* was deleted in 67% of cases. These results, obtained by gene copy dosage, are consistent with those deduced from earlier cytogenetic (Vogelstein *et al*, 1988; Mitelman *et al*, 1997) and loss of hetereozygocity (LOH) studies on the 18q21 region (Laurent-Puig *et al*, 1992; Jen *et al*, 1994; Martínez-López *et al*, 1998; Jernwall *et al*, 1999; Watanabe *et al*, 2001). However, these LOH studies are frequently based on microsatellite markers that span several centiMorgans (cM), and therefore several megabases (Mb). In contrast to that approach, our strategy allows for a more refined analysis by targeting an individual gene rather than a wide chromosomal region that certainly contains a number of important genes. Thus, it is likely that an analysis at the single gene level will give a more accurate image of eventual clinical implications of genetic alterations.

We observed that colorectal cancer patients with normal *SMAD4* gene copy status had a three-fold higher benefit of 5FU-based therapy than those with *SMAD4* deletion. This result is consistent with the previous observation by Watanabe *et al* (2001), that patients with retention of 18q21 alleles had a benefit of 5FU-based chemotherapy of the same order as was found in this study. Moreover, refinement of deletion studies from the chromosomal band level to the gene level may provide a clue to possible mechanisms through which 18q21 deletion influences the outcome of patients with CRC. Therefore, our results reinforce the hypothesis that TGFβ and its signalling components have a role in tumour suppression. This result also suggests the definition of *SMAD4* as a predictive marker for benefit of 5FU-based chemotherapy in patients with colorectal cancer. Finally, these findings suggests a mode of action of this cytostatic compound that is SMAD4-dependent. Thus, the integrity of this component of the TGFβ/BMP pathway is not only required for cytokine signalling, but may also be an important factor for 5FU-mediated apoptosis. In addition to the requirement of functional apoptotic pathways such as CD95/Fas (Houghton *et al*, 1997), bax (Rampino *et al*, 1997) and p53 (Vogelstein *et al*, 2000) for drug sensitivity in colorectal tumour cells, this suggests that integrity of the TGFβ pathway may be an additional condition for efficiency of 5FU treatment. Thus, our results provide an additional clue to the genetic basis of drug resistance in cancer.
