# SMAD4 is a predictive marker for 5-fluorouracil-based chemotherapy in patients with colorectal cancer

**DOI:** 10.1038/sj.bjc.6601020

**Published:** 2003-07-15

**Authors:** J-L Boulay, G Mild, A Lowy, J Reuter, M Lagrange, L Terracciano, U Laffer, R Herrmann, C Rochlitz

## Abstract

**Correction to**: British Journal of Cancer (2002) **87**, 630–634. doi:10.1038/sj.bjc.6600511

Unfortunately due to a typesetting error, Figure 2

**Figure 2:**
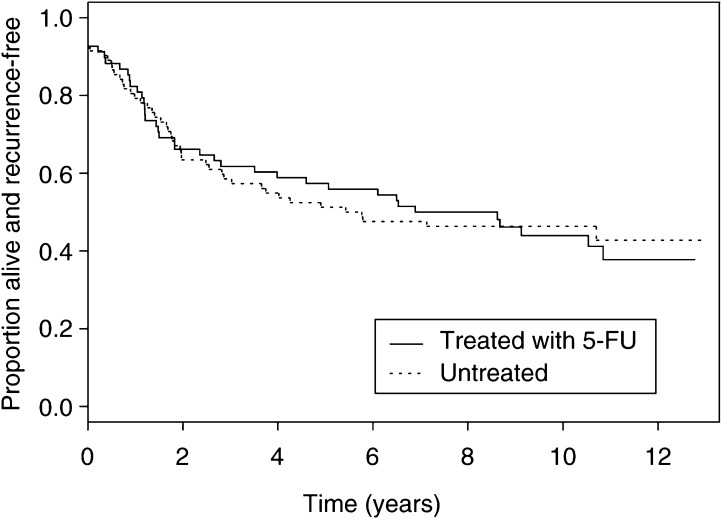
Disease-free survival by 5-FU treatment (SMAD4 deleted).

was reproduced uncorrectly.

The correct version is reprinted below:

The publisher would like to apologise for any inconvenience this may have caused.

